# Sustainability-aware user association for RIS-assisted mmWave networks in dense blockage environment

**DOI:** 10.1371/journal.pone.0354379

**Published:** 2026-08-04

**Authors:** Aisha Mumtaz, Sundus Ali, Muhammad Imran Aslam

**Affiliations:** 1 Department of Telecommunications Engineering, NED University of Engineering and Technology, Karachi, Pakistan; 2 Department of Computer Science, Bahria University Karachi Campus, Karachi, Pakistan; Nottingham Trent University School of Science and Technology, UNITED KINGDOM OF GREAT BRITAIN AND NORTHERN IRELAND

## Abstract

Millimeter wave (mmWave) technology offers vast bandwidth and ultra-high data rates but suffers from severe path loss and blockages. Reconfigurable intelligent surfaces (RISs) can mitigate the impact of blockages, but dense deployment of RISs adversely affect the overall energy requirement of the network. However, most of the existing work on RIS-assisted mmWave networks either assume passive RIS operation or rely on external power supply. The need to consider sustainable RIS operation in user association remains unaddressed, where sustainability refers to the RIS’s ability to operate continuously by harvesting energy from received RF signals without an external power source. This paper incorporates the concept of RIS energy sustainability into the user association probability of an RIS-assisted link. In this paper, a stochastic geometry based analytical framework for user association in a mmWave network assisted by self-sustainable RISs has been proposed. In addition, a baseline model for a conventional RIS-assisted link has also been developed that serves as a benchmark for comparison with the proposed model. Unlike conventional approach, each RIS in the proposed model operates in a self-sustainable manner. We have introduced a two-stage thinning process to identify RISs that are simultaneously in line-of-sight (LoS) with the user and capable of sustainable operation. Analytical expressions for RIS sustainability probability and user association probability have been derived, considering both direct, sustainable and conventional RIS-assisted communication links. We have performed a comparison of our proposed model with a learning based Deep Deterministic Policy Gradient (DDPG) model to validate the applicability of the proposed model for dense urban mmWave networks. The results obtained in this investigation reveal that increasing RIS deployment density significantly enhances the availability of sustainable LoS RISs, thereby improving the sustainability-aware user association probability and reducing outage in dense blockage environments. However, in highly dense environments, a tradeoff exists between increasing RIS deployment density to improve user association probability and ensuring self-sustainable RIS operation, thereby revealing a feasible RIS deployment region for sustainable operation. We demonstrate that, the proposed system remains fair and computationally inexpensive under varying network load, highlighting its applicability for large-scale and dense networks.

## I. Introduction

The extensive use of high-end applications and the exponential growth of mobile data traffic call for high data rates, low latency, and uninterrupted connectivity to ensure a seamless user experience. To address these challenges, robust communication systems must be designed. In this regard, extensive research is being carried out on sixth-generation (6G) networks to achieve very high data rates, ultra-low latency, massive capacity, low power consumption, and ubiquitous availability across terrestrial, aerial, spatial, and marine domains [1]. Prior works [2–7] have also explored broader aspects of next-generation wireless systems, including Reconfigurable Intelligent Surface (RIS)-assisted IoT security and backscatter communication, hybrid RIS architectures for secure transmission, and advanced resource allocation and spectrum sensing techniques. In this context, millimeter-wave (mmWave) spectrum has emerged as a powerful candidate for fulfilling the capacity requirements of beyond fifth-generation (5G) and 6G systems [2,7,8]. However, mmWave signals are vulnerable to obstacles that can lead to notable degradation in signal quality [9]. The key challenges of mmWave communications are severe path loss and vulnerability to blockages that require advanced technologies to achieve the desired network performance [10]. The integration of RISs in mmWave networks has become a transformative technology, revolutionizing wireless communication systems [11]. An RIS is an array of passive reflecting elements that can dynamically control the properties of signals incident upon it. By altering the amplitude or phase of the reflected waves, an RIS can effectively shape the wireless propagation environment, enabling enhanced communication performance [12]. The ability of RISs to manipulate the propagation environment results in a smart radio environment, making it a highly suitable option to meet the high availability demands of future wireless networks.

RISs have shown great potential to improve the system performance in the presence of blockages which greatly impacts the mmWave communication systems [13]. However, this performance improvement introduces new challenges in terms of increased energy requirements of the network, motivating the need for energy-efficient RIS designs [14,15]. Although RISs are considered passive devices since they do not actively generate RF signals, in practice they are not ideally passive [16,17]. Each reflecting element requires control power to adjust its phase shift. Moreover, the RIS panel relies on biasing circuits, Field Programmable Gate Array (FPGA) boards, and synchronization modules to maintain its operation [18]. The experimental results in [17] indicate that a 1000-element RIS requires a substantial amount of power to perform its operation. Similarly, a 15 × 15 dual-polarized RIS prototype has been shown to consume about 100 mW, along with static overhead arising from FPGA and driver circuits [19]. These studies challenge the conventional assumption of “completely passive” operation of RISs. Considering these practical power requirements, several studies have focused on improving energy efficiency in RIS-assisted system design. Study in [20] investigates the joint optimization of beamforming and RIS configurations under practical power constraints. In [21], the authors develop a framework for optimizing energy efficiency and resource allocation in wireless networks under quality of service constraints. Furthermore, the work in [3] proposes a UAV-assisted RIS-enabled framework for urban wireless powered communication networks. In parallel with these developments, the impact of the blockage conditions on the performance of RIS-assisted mmWave networks have been studied by different researchers. For instance, study in [22] addressed the impact of RIS on user association in multi base station mmWave systems. In [23], the user association probability is treated as an optimization problem. Authors in [24] address a joint user association problem to maximize the efficiency of a wireless network supported by multiple intelligent reflective surfaces (IRSs).

Existing hybrid beamforming approaches mainly focus on instantaneous optimization of active beamforming vectors and RIS phase shifts to maximize spectral efficiency or sum rate [25,26]. These methods rely on iterative procedures such as Block Coordinate Descent (BCD), manifold optimization (MO), Alternating Optimization (AO), Semidefinite Relaxation (SDR), Majorization–Minimization (MM), or Successive Convex Approximation (SCA) techniques [27,28]. The authors in [27] proposed a MO-BCD-based beam-pattern synthesis scheme for hybrid analog–digital arrays that jointly optimizes analog and digital beamformers using MO and BCD, enabling accurate beam shaping. The work in [28] proposes an adaptive zero-forcing maximum ratio combining (ZF–MRT) mixing strategy that dynamically balances interference suppression and beamforming gain based on real-time channel conditions. In [29], a mmWave radar-enabled RIS beamforming framework is presented, where radar-acquired user position information drives optimal phase configuration via an alternating direction method of multipliers (ADMM)-based distributed optimization. The authors in [30] proposed an alternating optimization algorithm based on manifold optimization (MOAO) to jointly optimize transmit beamforming and the phase-shift matrix of RIS for secrecy rate maximization. In addition to analytical and optimization-based approaches, recent research has explored the use of artificial intelligence (AI) and data-driven techniques for RIS-assisted wireless networks. The work in [31] uses Deep Reinforcement Learning (DRL) to jointly optimize BS beamforming and RIS phase shifts, achieving performance comparable to traditional methods. In [32], an attention-based Graph Neural Network (GNN) is used for joint beamforming and user association. By leveraging uplink pilots and graph-based learning, authors in [33] propose a joint optimization scheme for RIS-aided mmWave networks that satisfies strict load-balancing requirements. The work in [34] addresses the non-convex optimization of spectrum efficiency in multi-RIS downlink systems by employing a Deep Deterministic Policy Gradient (DDPG) algorithm. Unlike conventional models that assume independent phase and amplitude controls, authors in [34] incorporate a practical coupling effect between reflecting amplitude and phase shifts. The proposed framework optimizes precoding and RIS phase shifts based on instantaneous CSI. In [35], a two-stage ML-based algorithm for RIS-assisted mmWave multi-hop communications is proposed, where a GNN-based path scheduling algorithm is used followed by a DDPG-based joint optimization of active and passive beamforming. Table 1 compares the proposed framework with the existing beamforming approaches.

**Table 1:** Comparison table of existing beamforming techniques with the proposed model.

Technique	Advantage	Limitation
Riemannian Newton method (RNM)-enabled BCD [27]	Accurate beam shaping with limited RF-chains	High computational complexity due to iterative manifold optimization
RIS-Aided Adaptive Zero-Forcing and Maximum-Ratio Transmission Hybrid Precoding (RA-ZMHP) [28]	Interference mitigation with improved convergence of MMSE performance	Relies on iterative optimization and perfect CSI
ADMM-Based RIS Phase Optimization [29]	Improved SINR, Achievable rate and energy efficiency	Accurate tuning of ADMM penalty factor
Alternating optimization based on manifold optimization (MOAO) [30]	Improved secrecy performance under blocked direct link	Computational complexity due to iterative manifold optimization. Requires accurate CSI
GNN-based multi-hop path scheduling and DDPG-based joint active and passive beamforming [35]	Improved sum achievable rate	High CSI acquisition overhead in dynamic environments. Requires retraining
Sustainability-aware user association framework (Proposed in this paper)	Sustainability-aware user association probability in dense blockage. Low computational complexity, analytical tractability and scalability	Elementary beamforming is assumed. Adaptive techniques are not implemented.

In general, learning-based approaches are well-suited for highly dynamic environments characterized by time-varying channels, user mobility, and complex optimization spaces where accurate analytical modeling becomes challenging. Nevertheless, such methods typically require extensive training data and significant computational resources to maintain performance under varying network conditions. In contrast, model-based optimization techniques such as BCD, AO, MO, and ADMM are preferable when accurate CSI is available and near-optimal beamforming solutions are required without offline training. These methods offer deterministic optimization performance but incur high computational complexity due to iterative processing. For large-scale network-level analysis, where tractable performance analysis, scalability, low computational complexity, and theoretical insights are essential, analytical frameworks provide meaningful theoretical insight.

Accordingly, the practical advantage of the proposed sustainability-aware framework lies in its ability to analytically characterize feasible RIS participation and user association behavior in large-scale mmWave deployments without relying on optimization and training procedures. Moreover, unlike existing studies that assume continuously active RISs, the proposed framework directly incorporates RIS energy sustainability into the user association process, thereby providing a more realistic characterization of sustainable RIS-assisted network operation. While learning-assisted techniques may further improve adaptability in highly dynamic environments, the proposed analytical framework offers a scalable sustainability-aware approach with reduced computational complexity for network-level studies.

To address the inherent power consumption of RISs in an energy-aware perspective, recent research has shifted towards self-sustainable RIS operation. In this context, the concept of self-sustainable RIS operation has recently attracted attention as a means to realize fully autonomous reconfigurable surfaces. Recent contributions have explored the energy harvesting at RIS, where a portion of the impinging RF signal is rectified to provide continuous DC power to the surface electronics. Authors in [36] derived the sustainability condition for an energy-harvesting RIS by comparing the harvested power against the circuit power consumption. A hybrid RIS architecture is proposed in [37], where a subset of elements operate in energy-harvesting mode while remaining elements are dedicated to signal reflection.

Despite the emerging interest in sustainable RIS operation, much of the existing literature on RIS-assisted networks primarily focuses on energy harvesting at user devices. In contrast, studies that consider energy harvesting at the RIS are often limited to link-level analysis and do not fully extend to stochastic-geometry-based network modeling. Furthermore, these studies ignore the sustainability of the RIS in terms of user association and assume that RISs are always powered.

To the best of authors’ knowledge, no existing work has developed a stochastic geometry framework that integrates RIS energy harvesting constraints resulting in sustainability-aware user association criteria. Motivated by this gap, our analysis introduces a sustainability-aware user association framework. We have developed a stochastic geometry framework in which harvested energy at the RIS is used to determine RIS sustainability during the user association process. In contrast to AI-based methods, the proposed framework adopts a stochastic geometry approach to develop a tractable analytical model for sustainability-aware user association. This approach enables the characterization of the impact of spatial randomness, blockage effects, and energy harvesting constraints on RIS availability and association probability. Unlike learning-based approaches that treat the system as a black box, the proposed model reveals fundamental structural behaviors of the network that are difficult to extract from purely data-driven methods. Therefore, our analytical framework complements existing AI-based solutions by providing theoretical insights that can guide the design and deployment of RIS-assisted mmWave networks. Furthermore, unlike conventional RIS association frameworks based on the probability of LoS availability and blockage conditions [27,38], the incorporation of energy sustainability introduces a new decision-making challenge. Hence, the availability of an RIS becomes a function of its harvested energy, which depends on the BS-RIS link distance as well as on small-scale fading, whereas the usefulness of that RIS for communication depends on the RIS-UE link distance and blockage conditions. This results in a spatial dependency, where an RIS that is closer to the UE with high LoS probability may not be sustainable due to insufficient harvested energy, and conversely, a sustainable RIS closer to the BS may not provide a LoS path to the UE due to existence of a blockage.

Although the analytical tools employed for deriving harvested energy and sustainability probability are individually well-established, their integration under a sustainability-aware framework leads to novel system-level insights that cannot be captured by conventional RIS-assisted association schemes. Conventional RIS-assisted network models assume that RIS availability is independent of energy constraints. This assumption causes consistent improvement in association probability with increasing RIS density. In contrast, our framework reveals that when sustainability constraints are incorporated, this behavior fundamentally changes. The availability of a self-sustainable RIS is constrained not only by its spatial location but also its harvested energy. To model this behavior, we have adopted a sequential thinning approach applied to the PPP of RISs. RISs are first thinned based on RIS–UE LoS conditions, followed by a second thinning based on energy sustainability. This two-step thinning process ensures that only RISs that are both in LoS and energy-sustainable are considered for user association. Similar thinning-based approaches have been adopted in prior works for modeling spatial interactions and coverage in wireless networks [39–41]. Furthermore, the model characterizes a novel outage mechanism where communication failure may occur not only due to blockage but also due to the inability of RISs to sustain operation despite favorable propagation conditions. These structural behaviors are not observable in conventional RIS-assisted frameworks that neglect energy sustainability. Therefore, in our proposed model, the user association process is governed by a joint feasibility constraint that couples energy availability and link reliability across two independent spatial domains. The interplay of these two conditions introduces a new system-level bottleneck, where increasing RIS density leads to a tradeoff between RIS availability and sustainability instead of continuously improving association probability. As a result, this formulation enables the derivation of new expressions for the association probability that jointly capture the effects of network geometry, blockages, and energy sustainability of RIS. Our proposed analytical framework provides characterization of a sustainability-aware RIS-assisted mmWave network with energy harvesting criteria.

The key contributions of this work are summarized as follows:

- We show that the integration of energy harvesting constraints fundamentally reshapes the spatial availability of RISs. Unlike conventional models where RIS availability is governed by blockage conditions, the proposed framework reveals that the effective set of usable RISs is jointly determined by LoS probability and energy feasibility, resulting in reduced RIS availability region. In contrast to conventional RIS-assisted networks, we present the user association probability for sustainable RIS operation under energy harvesting constraints that exhibits a non-monotonic dependence on RIS deployment density. This fact reveals the existence of an optimal RIS deployment region.
- We identify a new outage mechanism arising from insufficient harvested energy at RISs, showing that communication failure may occur even in the presence of favorable propagation conditions. This leads to a modified outage scaling behavior compared to classical blockage-limited systems.
- We develop a modified stochastic geometry framework that jointly captures the impact of BS–RIS energy harvesting and UE–RIS propagation conditions, resulting in a novel tractable analysis of sustainability-aware user association.
- We introduce a two-stage thinning process based on LoS conditions between RIS and UE and harvested energy at RIS**.** The model analytically determines feasible RIS density for sustainability aware user association enabling scalable deployment of self-sustainable RISs in dense 28 GHz mmWave networks.
- We demonstrate that incorporating RIS sustainability constraint into the user association process maintains a fair distribution of user rates across the network while preserving analytical and computational tractability.

The paper is organized as follows. First, we elaborate the system model in section II. In section III, we develop the downlink received signal power expressions for the direct, conventional and sustainable RIS-assisted links. Section IV introduces the RIS energy harvesting model. In section V, we present the analytical framework for deriving the user association probability while incorporating the RIS energy harvesting and sustainability constraints. Section VI presents fairness analysis while section VII discusses system complexity. The analytical results are then presented and key insights are discussed in section VIII. Section IX provides the conclusion of the paper.

## II. System model

We consider the downlink of a multi-RIS assisted mmWave network consisting of one base station (BS), a typical user equipment (UE), L-number of RISs and randomly located blockages. BS is positioned at the center of the cell coverage area and is equipped with $N_{BS}$ antennas. Since wireless links are extremely sensitive to obstacles in mmWave band, frequent transitions from line-of-sight (LoS) to non-line-of-sight (NLoS) conditions are expected during communication. To capture these effects in our spatial model, we introduce a blockage process that characterizes the geometry and random placement of these obstacles. Therefore, buildings are modeled using a line Boolean blockage model [42] to statistically describe the random positions, orientations, and dimensions of the blockages. In the Boolean model of line segments, each blockage is represented by a line segment of random length and random orientation. The central positions of all line segments are modeled by a Poisson Point Process (PPP) $\Phi_{B}=\left\{z_{i}\right\}\in R^{\text{2}}$ with a density of $\lambda_{B}$. Here, $z_{i}$ is the position coordinate of the center of the $i^{th}$ blockage, and $\Phi_{B}$ denotes the set of center coordinates of all blockages. The length of each line segment is denoted as $L_{i}$ that follows a uniform distribution $U[L_{\text{min}},L_{\text{max}}]$ with a mean value of E[L]. Minimum and maximum lengths of all line segments, are denoted by $L_{\text{min}}$ and $L_{\text{max}}$ respectively. The orientation angle of each blockage, defined as the angle between the line segment and the positive direction of the x-axis, is assumed to follow a uniform distribution $\psi_{b,i}\sim U[\text{0},\text{2}\pi ]$.

**Fig 1:**
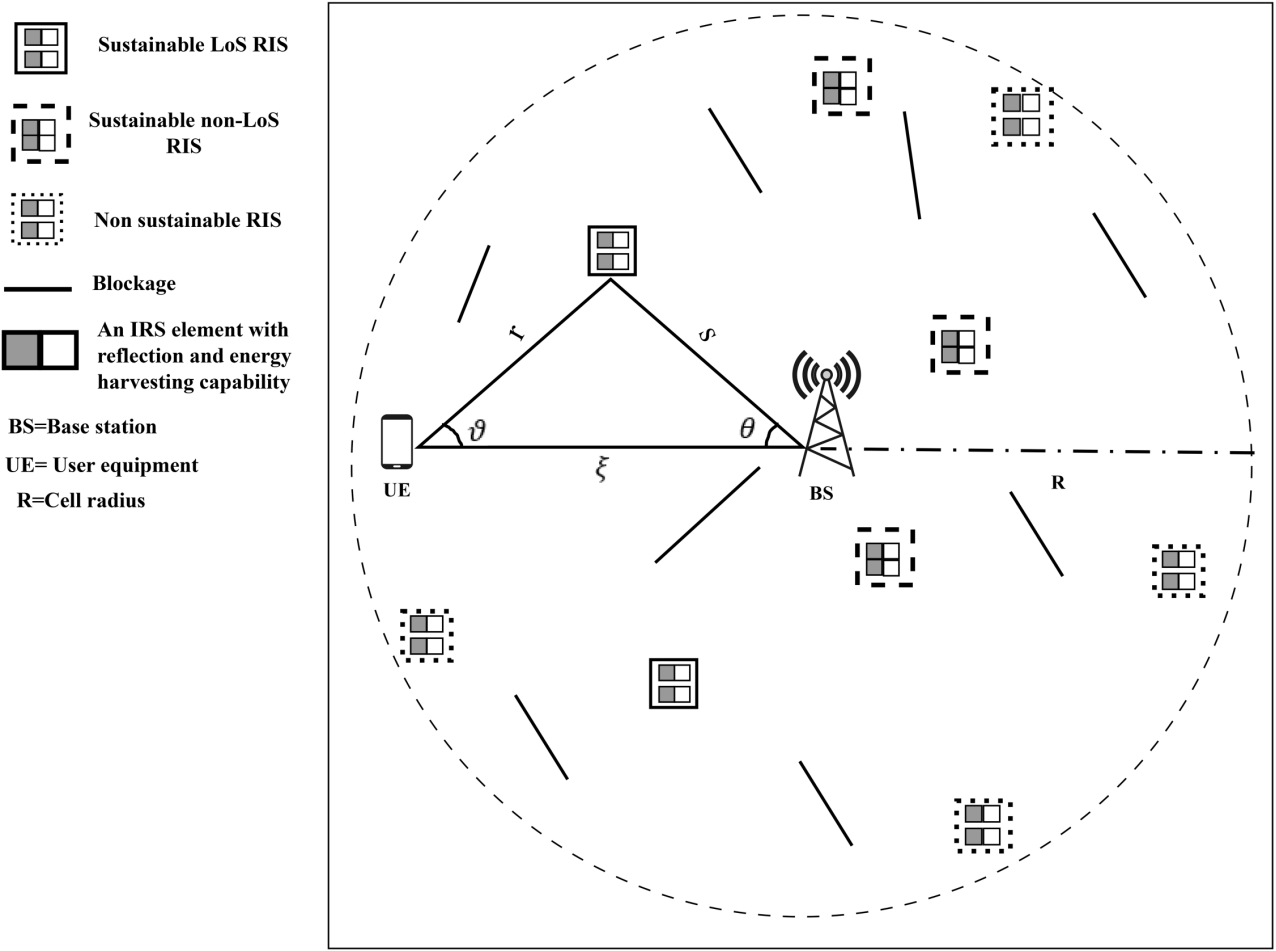
Illustration of RIS-assisted single-cell network showing sustainable and non-sustainable RISs.

We assume a typical UE equipped with $N_{u}$ antennas randomly located in the cell coverage area that represents the average performance of any user located within the cell coverage region. Since, according to Slivnyak’s theorem [43], introducing a user into a PPP at any arbitrary location does not alter the statistical properties of the remaining nodes. Therefore, the spatial behavior of the network observed at this typical UE is statistically equivalent to that of any other UE in the coverage region. We define ξ as the variable of the distance between BS and typical UE as illustrated in Fig 1. The RISs are spatially distributed according to a PPP denoted as $\Phi_{R}=\left\{R_{i}\right\}\in R^{\text{2}}$, with a density of $\lambda_{R}$, where $R_{i}$, $\Phi_{R}$, and N represent the position of the $i^{th}$

RIS, the set of all RIS coordinate points, and the number of RIS elements, respectively. The RIS is modeled as a uniform planar array consisting of $N=N_{x}\times N_{y}$ elements, where the horizontal and vertical inter-element spacing is set to $\frac{\lambda }{\text{2}}$, with λ denoting the signal wavelength [44]. The number of RIS elements N is determined by the physical size of the surface and the inter-element spacing. In this work, we assume a square RIS array of side length 0.6m resulting in RIS array size of 110 × 110 for 28 GHz. Each RIS element is equipped with a finite-resolution phase shifter and applies a discrete phase shift chosen from a codebook of size ${\text{2}}^{b}$, where b is the number of quantization bits as discussed in detail in [45].

Practically, an RIS is composed of sub-wavelength unit cells with tunable impedance. Each element can either reflect with a programmable phase or be connected to an RF rectifier to harvest energy. We assume a per-element power splitting model to represent the reflection/harvesting functionality of the RIS [46]. This model is physically feasible using a small RF splitter/combiner on a chip and is widely used for analytical tractability. Furthermore, it is assumed that the BS–RIS link is always line-of-sight (LoS), since RISs are strategically deployed with clear visibility of the BS. We assume that the small-scale fading of each RIS element follows a Rayleigh distribution. Accordingly, the per-element instantaneous channel power gain is exponential with mean 1. Under these assumptions, we derive the expression for the downlink received signal power through the direct link and RIS-assisted link in the next section.

## III. Downlink received signal power

In this section, we derive the downlink received signal power at a typical UE by considering both the direct link and the RIS-assisted link between the BS and typical UE. The analysis accounts for distance-dependent path loss, small-scale fading, and directional beamforming gains at the BS and the typical UE along with the reflection characteristics of the RIS.

### A. Downlink received signal power through the direct link

For a typical UE located at a distance ξ from the BS, the received signal power at the UE through the direct link, denoted as $S_{D}$, is expressed as


$$\begin{array}{c} S_{D}=g_{D}P_{t}h_{D} \end{array}$$ (1)

where $g_{D}$ is the large-scale fading channel gain of the direct link and is given as $g_{D}=\frac{{\text{1}\text{0}}^{\alpha }}{\xi^{\beta }}$. The $g_{D}$ follows 3GPP model where α is the reference path loss at 1m [47], and the parameter β represents the exponent of path loss. $P_{t}$ denotes the BS transmit power to each UE, and $h_{D}$ denotes the small-scale fading channel gain of the BS-UE link, which is modeled as an exponential random variable as $h_{D}\sim exp(\text{1}/\omega \;)$ with a mean value of ω where ${\omega =N}_{BS}\cdot N_{u}$. Hence $h_{D}$ can be denoted as $\begin{array}{c} h_{D}\sim exp(1/N_{BS}\cdot N_{u}) \end{array}$ [48]. This formulation captures the effective channel gain under beam-aligned transmission, where the combined antenna array gain $N_{BS}\cdot N_{u}$ scales the mean of the fading distribution. The data transmission link between the BS and the user is assumed to be implemented using existing beamforming techniques [49]. Given the direct link communication scenario, we proceed to characterize the downlink received signal power through the RIS-assisted link.

### B. Downlink received signal power through RIS-assisted link

In the case where the typical UE connects to the BS through the $l^{th\;}\;RIS$, we define the link as an RIS-assisted link. The received power of the signal through RIS-assisted link is given by


$$\begin{array}{c} S_{{RIS}_{l}}=P_{t}g_{l}\Theta_{l_{n}}^{disc}h_{BR_{l}U} \end{array}$$ (2)

where $h_{BR_{l}U}=h_{BR_{l}}.h_{R_{l}U}$, is the cascaded channel from BS to UE via $l^{th}$RIS and $h_{BR_{l}}=\left|h_{BR_{l}}\right|e^{j\varphi_{{BR}_{l}}}$ and $h_{R_{l}U}=\left|h_{R_{l}U}\right|e^{j\varphi_{R_{l}U}}$ represent the small scale fading channel gain from $BS-RIS_{l}$ and $RIS_{l}\;-UE$ respectively. Since small-scale fading coefficients of the $BS-RIS_{l}$ and $RIS_{l}\;-UE$ links are modeled as independent Rayleigh random variables, magnitudes of $h_{BR}$ and $h_{RU}$ follow exponential distribution such that $\begin{array}{c} h_{BR_{l}}\sim exp(1/N_{BS}\cdot N) \end{array}$ and $\begin{array}{c} h_{R_{l}U}\sim exp(1/N\cdot N_{u}) \end{array}$ representing the total received power after coherent combining across antennas and reflecting elements.. The large-scale fading channel gain for $l^{th}$ RIS-assisted link is denoted by $g_{l}$ and is given as $g_{l}\;=\frac{{\text{1}\text{0}}^{\text{2}\alpha }}{{\left({\text{s}}_{l}{\text{r}}_{l}\right)}^{\beta }}$. Here, ${\text{s}}_{l}$ and ${\text{r}}_{l}$ denote the distance of the $BS-RIS_{l}$ link and the $RIS_{l}\;-UE$ link respectively. The phases $\varphi_{BR_{l}}$ and $\varphi_{R_{l}U}$ are uniformly distributed between [0,2π). Thus, the phase shift matrix of $l^{th\;}\;RIS$ is defined as


$$\begin{array}{c} \begin{array}{c} \emptyset_{l_{n}}=diag\left\{\emptyset_{l_{\text{1}}},\emptyset_{l_{\text{2}}},\ldots .,\emptyset_{l_{N}}\right\} \end{array} \end{array}$$ (3)

where $\emptyset_{l_{\text{n}}}$ is the phase shift introduced by the $n^{th}$ element of the $l^{th}$ RIS. We define $\emptyset_{l_{\text{n}}}^{opt}$ as the optimal phase shift provided by each RIS element such that


$$\begin{array}{c} \begin{array}{c} \emptyset_{l_{\text{n}}}^{opt}=-\varphi_{{BR}_{l}}-\varphi_{R_{l}U} \end{array} \end{array}$$ (4)

where $\varphi_{{BR}_{l}}$and $\varphi_{R_{l}U}$ are the phases of the channels from BS to the $n^{th}$ element of the $l^{th}$ RIS and from $n^{th}\;$element of the$\;l^{th}\;RI\text{S }$ to the UE, respectively. This optimal phase shift has to be quantized to the nearest discrete value. We assume that the discrete phase shift introduced by each RIS element can take only a finite number of discrete values. Thus, the set of discrete phase shift values introduced by the $l^{th}$ RIS is given as


$$\begin{array}{c} \begin{array}{c} \Theta_{l_{n}}^{disc}=diag\left\{\theta_{l_{\text{1}}}^{disc},\theta_{l_{\text{2}}}^{disc},\ldots .,\theta_{l_{N}}^{disc}\right\} \end{array} \end{array}$$ (5)

where $\theta_{l_{n}}^{disc}=\Delta \theta .round(\frac{\emptyset_{l_{n}}^{opt}}{\Delta \theta })$and $\Delta \theta =\frac{\text{2}\pi }{{\text{2}}^{b}}$. The discrete phase shift design ensures practical implementation of the RIS reflection directly influencing the amount of RF energy available for harvesting, which is analyzed in the next section.

## IV. RIS energy harvesting model

To enable self-sustained operation at the RIS for achieving sustainable network deployment goals, each RIS is assumed to be equipped with an energy harvesting circuit that converts a portion of the incident RF energy into usable DC energy. This harvested energy supports the control circuitry and reflection operations of the RIS, thereby eliminating the need for external power source. The self-sustainable operation of RIS facilitates dense urban environments, where wired power or frequent battery replacement is impractical. Since each RIS has N reflecting elements, when the BS serves the typical UE through the $l^{th}\;RIS$, the total instantaneous per element RF power incident on the $l^{th}$ RIS is defined as the power incident on the $n^{th}$ element of the $l^{th}\;$RIS through the BS and is given by


$$\begin{array}{c} \begin{array}{c} p_{b,l_{n}}=P_{t}g_{s_{l}}G_{BF}X_{l_{n}} \end{array} \end{array}$$ (6)

where $g_{s_{l}}=\frac{{\text{1}\text{0}}^{\alpha }}{{s_{l}}^{\beta }}$, we define $s_{l}$ as the distance between BS and the $l^{th}\;RIS$. $X_{l_{n}}\sim exp(\text{1})$ and $G_{BF}$ is the beamforming gain assuming perfect phase alignment across all transmit antennas [50]. In practical RIS-assisted mmWave systems, the beamforming gain is limited by inaccuracies in the channel estimation [51,52] which can be reflected in terms of CSI quality factor (τ∈[0,1]). In this situation, the effective beamforming gain (${\text{G}}_{\text{eff}}$) is generally modelled as $G_{eff}=\tau^{\text{2}}\times G_{BF}$. The case τ=1 corresponds to perfect CSI, whereas smaller values of τ represent increasing channel estimation errors due to imperfect CSI. This formulation captures the performance loss caused by imperfect CSI in practical RIS-assisted mmWave deployments. Hence, the total incident power on the $l^{th}$ RIS can be expressed as the sum of the received powers across all elements and is given by


$$\begin{array}{c} P_{inc,l}=\sum_{n=\text{1}}^{N}p_{b,l_{n}}=P_{t}g_{s_{l}}G_{eff}\;\sum_{n=\text{1}}^{N}X_{l_{n}} \end{array}$$ (7)

Thus, the energy harvested at the $l^{th}$ RIS is given by


$$\begin{array}{c} \begin{array}{c} E_{harv,l}=\rho \epsilon TP_{inc,l} \end{array} \end{array}$$ (8)

where ϵ∈(0,1) is the per element power splitting ratio [53], *T* is the time slot during which the channel is assumed to be quasi-static [54] and ρ is the rectifier efficiency as discussed in detail in references [55,56].The parameter ρ accounts for the nonlinear energy conversion losses in the RF-to-DC conversion process at the RIS energy harvesting circuit**.** In this work, energy harvesting system is assumed to be ideal that maintains constant efficiency across all power levels with ignorable nonlinearities, hence the parameter ρ is treated as constant. Therefore, equation (8) represents that the harvested energy is linearly related to the power incident on the RIS as given in (7). This linear energy harvesting model provides a tractable mathematical framework to analyze the energy harvesting performance of RIS [55].

### A. RIS sustainability model

An RIS is said to be sustainable if the energy harvested by the RIS is greater than its total energy consumption, which includes the energy required for the controller, phase shifters, and circuit power. This condition ensures that the RIS can operate independently without external energy sources and maintains its functionality in energy-constrained networks. Now we derive the sustainability probability of the RIS as follows


$$\begin{array}{c} \begin{array}{c} P_{sust}(s)=\text{Pr}\left(E_{harv}>E_{RIS}\right) \end{array} \end{array}$$ (9)

where $E_{RIS}$ is the total energy required by the RIS to sustain its operation and is given as [50]


$$\begin{array}{c} E_{RIS}={(P}_{c}+NP_{n}(b))T \end{array}$$ (10)

where $P_{n}(b)$ is the power consumption of each phase shifter having b-bit resolution and $P_{c}$ is the power consumption of the RIS controller circuit [21].


$$\begin{array}{c} P_{sust}(s)=\text{Pr}\left(\rho \epsilon P_{t}TG_{eff}g_{s_{l}}\sum_{n=\text{1}}^{N}X_{l_{n}}>E_{RIS}\right) \end{array}$$ (11)

$$\begin{array}{c} P_{sust}(s)=\text{Pr}\left(\sum_{n=\text{1}}^{N}X_{l_{n}}>\frac{E_{RIS}}{\rho \epsilon P_{t}{TG}_{eff}g_{s_{l}}}\right) \end{array}$$ (12)

Substituting $g_{s_{l}}=\frac{{\text{1}\text{0}}^{\alpha }}{{s_{l}}^{\beta }}$ in (12), we get


$$\begin{array}{c} P_{sust}(s)=\text{Pr}\left(\sum_{n=\text{1}}^{N}X_{l_{n}}>\frac{E_{RIS}{s_{l}}^{\beta }}{\rho \epsilon P_{t}TG_{eff}{\text{1}\text{0}}^{\alpha }}\right) \end{array}$$ (13)

Let $Y_{n}=\sum_{n=\text{1}}^{N}X_{l_{n}}$ and $\delta =\;\frac{E_{RIS}{s_{l}}^{\beta }}{\rho \epsilon P_{t}{TG}_{eff}{\text{1}\text{0}}^{\alpha }}$ we can write


$$\begin{array}{c} \begin{array}{c} P_{sust}(s)=\text{Pr}\left(Y_{n}>\delta \right) \end{array} \end{array}$$ (14)

Since $Y_{n}$ is a Gamma random variable with shape N and scale 1, its pdf is $f_{Y}(y)=\frac{y^{N-\text{1}}\text{exp}(-y)}{(N-\text{1})!},\;y\geq \text{0}$ [57]. Therefore, the harvested energy at RIS is modeled as a Gamma-distributed random variable and RIS sustainability probability is a tail probability which means that it represents the likelihood that the harvested power at the RIS exceeds a required threshold. Therefore, it is calculated using upper incomplete Gamma function and is given as


$$\begin{array}{c} \begin{array}{c} P_{sust}(s)=\int_{\delta }^{\infty }\frac{y^{N-\text{1}}\text{exp}(-y)}{(N-\text{1})!}dy \end{array} \end{array}$$ (15)

Since $\Gamma (a,x)=\int_{\delta }^{\infty }t^{a-\text{1}}e^{-t}dt$, we can write


$$\begin{array}{c} \begin{array}{c} P_{sust}(s)=\frac{\Gamma (N,\delta )}{(N-\text{1})!} \end{array} \end{array}$$ (16)

For integer N, the upper incomplete Gamma reduces to a closed-form finite sum. Hence, we can write


$$\begin{array}{c} \Gamma (N,\delta )=(N-\text{1})!\;\text{exp}(-\delta )\sum_{k=\text{0}}^{N-\text{1}}\frac{\delta^{k}}{k!} \end{array}$$ (17)

Substituting (17) into (16), we get


$$\begin{array}{c} P_{sust}(s)=\text{exp}(-\delta )\sum_{k=\text{0}}^{N-\text{1}}\frac{\delta^{k}}{k!} \end{array}$$ (18)

Thus, (18) provides exact form of the sustainability probability of an RIS. It quantifies how often an RIS remains self-sustainable and hence available for reflection. As a result, this energy-feasibility requirement dictates the association criteria detailed in the following section.

## V. User association probability

User association probability plays an important role in determining the performance of RIS-assisted mmWave networks as it governs how a typical UE selects its serving link. Accordingly, user association is determined by predefined association criteria that determines whether the UE connects via the direct BS–UE link or an RIS-assisted link. Based on this criterion, the corresponding user association probabilities for the direct and RIS-assisted links are derived. In this system model, we consider distance-dependent user association criteria, where a typical UE selects the link that offers the minimum effective propagation distance. Based on this criteria, the user association probabilities for the direct BS–UE link and the sustainable and conventional RIS-assisted links are analytically derived.

### A. User association criteria

**Fig 2:**
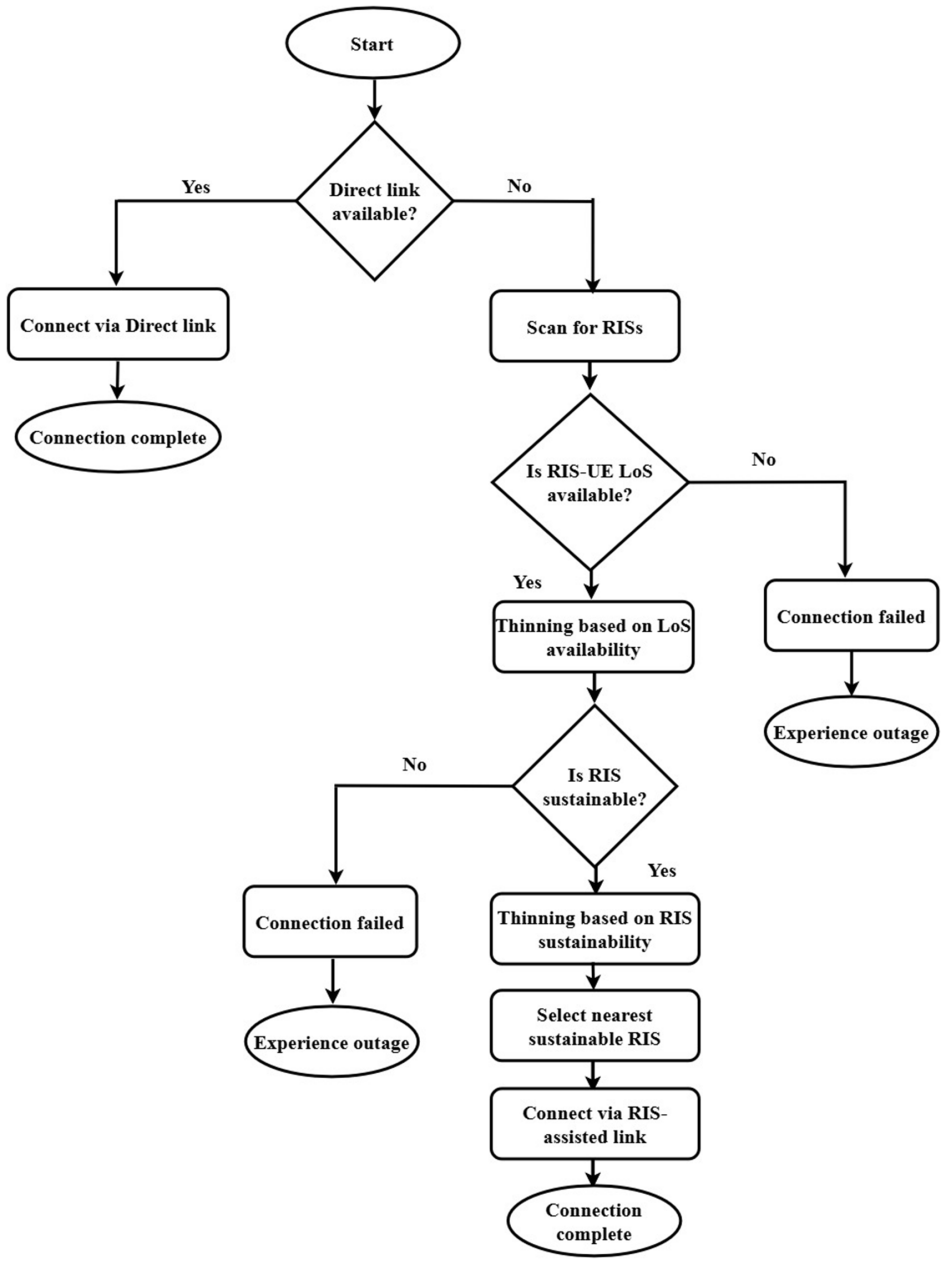
User association scheme for sustainable RIS-assisted mmWave network.

Number of different user association scenarios can emerge in a propagation environment with randomly located RISs and blockages. A typical UE connects to the serving BS directly, through the RIS-assisted path or it may experience an outage. Given that the multiplicative path loss of the RIS reflection link is significantly higher than that of the direct link, the association criteria will simplify. Specifically, the user first attempts to establish a direct link with the BS whenever an LoS connection with the BS exists. When the direct link is blocked, out of all the RISs available in the vicinity of the UE, some RISs might not be able to perform sustainable operation and some of them might be blocked. We define the inhomogeneous PPP of sustainable LoS RISs available for association as $\Phi_{R}^{S}$, explained in detail in section V-D. In case of an RIS-assisted link, the UE connects to the BS through the nearest sustainable LoS RIS. Under this association scheme, the criteria for a direct link is implicit while the criteria for selecting the associated RIS is defined as $l^{*}\;:=\underset{r_{l}\in \Phi_{R}^{S}}{\text{min}}r_{l}$. If both the LoS and sustainable RIS-assisted links are not available, UE experiences an outage. Fig 2 illustrates the user association process and resulting association decision based on the availability of direct or RIS-assisted link.

Based on the defined user association criteria, we now derive the corresponding user association probabilities for the direct, conventional and sustainable RIS-assisted links.

### B. User association probability for direct link

In a mmWave network with blockages, there is a chance for the typical user to acquire a direct path to connect with the nearest BS. However, the probability of this event depends upon the average density of blockages present in the network. Hence, the probability of the existence of a LoS link at a distance *d* is is given as [58]


$$\begin{array}{c} \begin{array}{c} P_{LoS}(d)=\text{exp}\left(-\frac{\text{2}\lambda_{B}E[L]}{\pi }d\right) \end{array} \end{array}$$ (19)

where d is the distance between the typical UE and the serving BS, and $E[L]=\frac{l_{\text{min}}+l_{\text{max}}}{\text{2}}$ is the expected length of a line segment blockage. Therefore, the direct user association probability given that the distance between typical UE and the BS is ξ, can be written as


$$\begin{array}{c} \begin{array}{c} P_{d}(\xi )=P_{LoS}(\xi ) \end{array} \end{array}$$ (20)

This expression implies that a user is directly associated with the BS only if a LoS link exists depending upon the distance of the user from BS, length of blockages and average density of blockages in the network.

### C. User association probability for conventional RIS-assisted link

In a practical scenario of an RIS-assisted mmWave network, not all RISs have sufficient harvested energy to assist the UE at a given time, due to variations in incident RF power, energy consumption and blockages. As a result, only a subset of RISs participates in sustainability-aware user association, while the remaining RISs are inactive from a sustainability perspective. To analytically capture this behavior, we first develop a baseline model based on conventional RIS operation, where all RISs are assumed to be “always-on” without energy constraints. The term “always-on RISs” refers to RISs that are available for communication and are assumed to operate without energy harvesting limitations. This baseline serves as a reference for evaluating the proposed energy-sustainability-aware user association strategy. A well-known property of PPPs is that the independent thinning of a PPP preserves the Poisson property. As long as each point is independently retained or removed with some probability, the resulting point process is still a PPP [59]. For the comparison of sustainable LoS RISs with the conventional model we define the set of conventional RISs as $\Phi_{R}^{Conv}$. Under the conventional RIS assumption, thinning is performed based only on the LoS availability between the RIS and the typical UE. The PPP of RISs, $\Phi_{R}$ with density $\lambda_{R}$ is first thinned with the LoS probability $P_{LoS}(r)$, where r is the distance from the typical UE to the RIS. The resulting inhomogeneous PPP of LoS RISs is denoted as, $\Phi_{R}^{Conv}$ with a density of


$$\begin{array}{c} \lambda_{R}^{Conv}(s,\theta ;\xi )=\lambda_{R}.P_{LoS}(r(s,\theta ,\xi )) \end{array}$$ (21)

We introduce (r,ϑ) as user-centric polar coordinates and (s,θ) as BS-centric polar coordinates as illustrated in Fig 1. We define ϑ as the angle between the UE-BS link and the UE-RIS link and θ as the angle between BS-UE and BS-RIS link. Referring to Fig 1 and utilizing law of cosines, $r=\sqrt{s^{\text{2}}+\xi^{\text{2}}-\text{2}\xi scos(\theta )}$. This distance relationship plays an important role in determining the received power at the RIS surface and consequently its energy harvesting capability. To find the expected number of conventional RISs around the BS, we integrate (21) over the area using BS-centered polar coordinates (s,θ). Hence, the average number of conventional RISs in the cell coverage area that can provide a LoS link to the user is given by


$$\begin{array}{c} \begin{array}{c} R^{Conv}(\xi )=\int_{\text{0}}^{\text{2}\pi }\int_{\text{0}}^{R}\lambda_{R}^{Conv}(s,\theta ;\xi )sdsd\theta \end{array} \end{array}$$ (22)

$$\begin{array}{c} \begin{array}{c} R^{Conv}(\xi )=\lambda_{R}\int_{\text{0}}^{\text{2}\pi }\int_{\text{0}}^{R}P_{LoS}\left(r(s,\theta ,\xi )\right)sdsd\theta \end{array} \end{array}$$ (23)

Hence, the probability that at least one conventional RIS exists in the cell coverage area follows from the void probability of PPP as


$$\begin{array}{c} \begin{array}{c} P_{Conv}(\xi )=\text{1}-\text{exp}\left(-R^{Conv}(\xi )\right) \end{array} \end{array}$$ (24)

Therefore, the user association probability for a conventional RIS-assisted link when the LoS is blocked becomes


$$\begin{array}{c} \begin{array}{c} P_{Conv-RIS}(\xi )={(\text{1}-P}_{LoS}(\xi ))P_{Conv}(\xi ) \end{array} \end{array}$$ (25)

This expression serves as the baseline model for our network. In the next section, we derive the energy-aware user association probability for sustainable RIS-assisted link.

### D. User association probability for sustainable RIS-assisted link

In contrast to conventional RISs, sustainable RISs are capable of harvesting sufficient energy for their operation, enabling them to assist the UE without the need for any external power source. This allows us to model the user association probability considering only the subset of RISs that can reliably sustain their operation. In order to obtain the set of LoS and sustainable RISs $\Phi_{R}^{S}$, a two-step thinning process is applied. The PPP of RISs, $\Phi_{R}$ with density $\lambda_{R}$ is first thinned with the LoS probability $P_{LoS}(r)$, where r is the distance from the typical UE to the RIS. We call the resulting inhomogeneous PPP of LoS RISs, $\Phi_{R}^{L}$ with a density of $\lambda_{R}^{L}=\lambda_{R}.P_{LoS}(r)$. In the second step,$\Phi_{R}^{L}$, is thinned with the probability of sustainable RISs, $P_{sust}(s)$ where s is the distance of the RIS from the BS. The events $P_{LoS}(r)$ and $P_{sust}(s)$ are independent events for each RIS. Therefore, the final thinned process remains a PPP, but with a new, location-dependent density that is the product of the original density and the two thinning probabilities. The resulting inhomogeneous PPP of “sustainable LoS RISs” $\Phi_{R}^{S}$ will have a combined density that depends on both r and s given as


$$\begin{array}{c} \begin{array}{c} \lambda_{R}^{S}(s,\theta ;\xi )=\lambda_{R}.P_{LoS}\left(r(s,\theta ,\xi )\right)P_{sust}(s) \end{array} \end{array}$$ (26)

To find the expected number of sustainable LoS RISs around the BS, we integrate (26) over the area using BS-centered polar coordinates (s,θ). Hence, the average number of sustainable RISs in the cell coverage area that can provide a LoS link to the user is given by


$$\begin{array}{c} \begin{array}{c} R^{S}(\xi )=\int_{\text{0}}^{\text{2}\pi }\int_{\text{0}}^{R}\lambda_{R}^{S}(s,\theta ;\xi )sdsd\theta \end{array} \end{array}$$ (27)

$$\begin{array}{c} \begin{array}{c} R^{S}(\xi )=\lambda_{R}\int_{\text{0}}^{\text{2}\pi }\int_{\text{0}}^{R}P_{LoS}\left(r(s,\theta ,\xi )\right)P_{sust}(s)sdsd\theta \end{array} \end{array}$$ (28)

where R is the cell radius. Equation (28) is evaluated from its analytical integral form using numerical quadrature to maintain analytical tractability [59,60]. Hence, the probability that at least one sustainable LoS RIS exists in the cell coverage area follows from the void probability of PPP as


$$\begin{array}{c} \begin{array}{c} P_{S}(\xi )=\text{1}-\text{exp}\left(-R^{S}(\xi )\right) \end{array} \end{array}$$ (29)

Therefore, the sustainability-aware user association probability for RIS assisted link when the LoS is blocked becomes


$$\begin{array}{c} P_{S-RIS}(\xi )={(\text{1}-P}_{LoS}(\xi ))P_{S}(\xi ) \end{array}$$ (30)

When the UE is positioned at a considerable distance from the BS and experiences a highly dense blockage environment, the direct and RIS-assisted links become vulnerable to severe degradation. Hence, the outage probability must be taken into account. We define the outage probability of a UE at a distance ξ from the BS as


$$\begin{array}{c} \begin{array}{c} P_{outage}(\xi )={\text{1}-P}_{LoS}(\xi )-P_{S-RIS}(\xi ) \end{array} \end{array}$$ (31)

This metric quantifies the likelihood that the UE experiences a communication failure due to unfavorable link conditions. It also provides key insight into the network reliability under varying propagation environments.

## VI. Fairness Analysis

In a multi-user network, it is important to assess how fairly communication resources are distributed among users. Since fairness is evaluated based on the achievable rates of UEs obtained through either direct or S-RIS-assisted communication, therefore to determine these rates, the received signal quality is first characterized through the Signal-to-Noise Ratio (SNR) for direct and RIS-assisted paths respectively.

For a UE served through the direct link, we represent the SNR of the $k^{th}$ UE served by direct link as


$$\begin{array}{c} \begin{array}{c} \Upsilon_{\text{D},\text{k}}=\frac{S_{D}}{\sigma^{\text{2}}} \end{array} \end{array}$$ (32)

where $S_{D}$ is given by (1) and the term $\sigma^{\text{2}}$ represents the noise power at the receiver.

Similarly, we characterize the SNR of the $k^{th}$ UE served by the $l^{th}$ S-RIS as


$$\begin{array}{c} \begin{array}{c} \Upsilon_{{S-RIS}_{l},\text{ k}}=\frac{S_{{S-RIS}_{l}}}{\sigma^{\text{2}}} \end{array} \end{array}$$ (33)

Substituting eq (2) in eq (33), we get


$$\begin{array}{c} \begin{array}{c} \Upsilon_{{S-RIS}_{l},k}=\frac{P_{t}{\text{1}\text{0}}^{\text{2}\alpha }\Theta_{l_{n}}^{disc}h_{BRU}}{\sigma^{\text{2}}{\left(s_{l}.r_{k}\right)}^{\beta }} \end{array} \end{array}$$ (34)

Where $r_{k}$ is the distance between $l^{th}$ RIS and the $k^{th}$ UE. Based on the obtained SNR for the direct and RIS-assisted links, the achievable rate of the $k^{th}$ UE is calculated as


$$\begin{array}{c} \begin{array}{c} @c{\text{R}}_{\text{k}}=\left\{\begin{array}{c} @cBW\;{\text{log}}_{\text{2}}\left(\text{1}+\Upsilon_{\text{D},\text{k}}\right),\;if\;direct\;link\;is\;available\\ BW\;{\text{log}}_{\text{2}}\left(\text{1}+\Upsilon_{{S-RIS}_{l},k}\right),\;if\;direct\;link\;is\;blocked,\;S-RIS\;is\;available\\ \text{0},\;if\;direct\;link\;is\;blocked\;and\;S-RIS\;link\;is\;blocked \end{array}\;\;\;\right\} \end{array} \end{array}$$ (35)

where B is the system bandwidth. Using the achievable rates of all served UEs, we evaluate user-rate fairness through JFI that is a widely adopted metric for assessing the uniformity of resource allocation in wireless networks [61]. Therefore, the fairness index is defined as


$$\begin{array}{c} \begin{array}{c} \text{JFI}=\frac{{\left(\sum_{i=\text{1}}^{K}R_{k}\right)}^{\text{2}}}{K\sum_{i=\text{1}}^{K}{R_{k}}^{\text{2}}} \end{array} \end{array}$$ (36)

where *K* denotes the total number of served UEs and $R_{k}$ represents the achievable rate of the $k^{th}$ UE. A value of JFI = 1 indicates perfect fairness, meaning that all UEs achieve identical data rates, whereas lower values correspond to increasing differences in UE rates. In the proposed sustainability-aware RIS-assisted framework, fairness is influenced by several factors, including UE locations, blockage conditions, sustainable RIS availability, and the quality of the direct and RIS-assisted communication links.

## VII. System complexity

The sustainability probability of an RIS, $P_{sust}(s)$, UE association probability through direct link,$P_{d}(\xi )$, and sustainable RIS association probability, $P_{S-RIS}(\xi )$ given in (18), (20) and (30) respectively are each evaluated in closed form with O(1) cost per user and contribute negligibly to the overall complexity. Although the calculation of $R^{S}(\xi )$ in (28) requires numerical quadrature over a two-dimensional integration domain of $N_{s}\times N_{\phi }$ points, contributing $O{(N}_{s}\times N_{\phi })$ per evaluation of $P_{S-RIS}(\xi )$. However, since $N_{s}$ and $N_{\phi }$ are fixed discretisation parameters chosen independently of *K* and $\lambda_{S-RIS}$, this quadrature cost is absorbed into a constant pre-computation term and does not affect the scaling behavior of the framework. Hence, the computational cost is dominated by the search for nearest S-RIS, that requires calculation of the Euclidean distance between each of the *K* users and each deployed S-RIS. As a result, the computational complexity of proposed framework is expected to scale linearly with both the number of users and the sustainable RIS density. The nearest RIS search incurs a complexity of ${O(K\cdot M}_{S-RIS})$ where $M_{S-RIS}=\lambda_{S-RIS}\pi R^{\text{2}}$ denotes the number of sustainable RISs within the cell coverage area. In addition, the SNR and rate computation for each served user requires O(1) arithmetic operations per user, contributing O(K) in total. Since $M_{S-RIS}\geq \text{1}$, it follows that ${K\cdot M}_{S-RIS}\geq K$. Therefore, the O(K) complexity of SNR and rate computation is also dominated by the ${O(K\cdot M}_{S-RIS})$ complexity of the nearest S-RIS search and can be absorbed into the dominant term. Consequently, these additional computations do not affect the overall complexity. Therefore, the computational complexity of the proposed sustainability-aware RIS-assisted mmWave single-cell network is given by ${O(K\cdot M}_{S-RIS})$. This computational complexity is substantially lower than the existing optimization and learning based approaches that rely on iterative optimization or training and inference resulting in a quadratic or cubic dependence on the system dimensions [27,28,35].

## VIII. Results and discussion

This section presents results for analytical expressions derived for sustainable and conventional RIS-assisted user association probability in terms of important system parameter such as association probabilities, outage probability, and harvested energy. We study the relationship between different system parameters and their influence on system performance, highlighting several key insights. We have compared the results for different blockage and RIS densities to analyze the performance of our sustainability-aware RIS association scheme for a randomly located user in the cell coverage area. We assume that the mmWave network operating frequency is $f_{c}=\text{2}\text{8}\;GHz$. The large-scale fading is characterized by the path-loss exponent =2.2, which determines how the received power decays with distance. The average length of blockage is set to E[L]=15 m. We assume that BS is equipped with $N_{BS}=\text{1}\text{2}\text{8}$ antennas and has a transmit power of 2 W. Radius of the cell is set to R=100 m. Each RIS is equipped with a 110×110 array of elements. The RIS has a per element power splitting ratio of ϵ=0.5. The power splitting ratio is fixed because in our proposed system model it acts as a scaling factor for the harvested energy as it indirectly influences RIS participation through the energy sustainability condition. This modeling approach is consistent with recent works where the power splitting ratio is fixed to isolate the impact of RIS design and system parameters. In most existing studies, power splitting is applied at the receiver [62,63], where it directly affects the trade-off between information decoding and energy harvesting. The splitting factor ϵ controls this division, where ϵ represents the portion of signal power used for energy harvesting, and 1−ϵ represents the portion of the signal power that could not be harvested [64,65]. The energy harvesting circuit is assumed to have a rectifier efficiency of ρ=0.7. The power consumption of RIS controller is $P_{c}=\text{1}\text{9}\;mW$. We assume perfect CSI and hence τ=1 for all the results presented in this work. However, we have also presented the impact of imperfect CSI for different values of τ.

**Fig 3:**
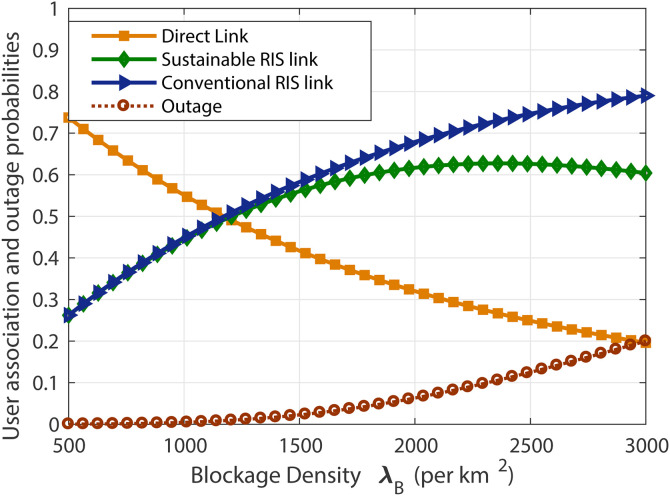
Direct, sustainable and conventional RIS-assisted user association and outage probabilities vs. blockage density $\lambda_{B}$ for RIS density $\lambda_{R}=1000$ per km^2.^

In practical RIS implementations, the total power consumption consists of a static component associated with the control circuitry and a dynamic component related to the individual elements. Prior works have shown that the controller and biasing circuits introduce a fixed baseline power consumption that is largely independent of the instantaneous configuration and channel conditions [56,58]. Therefore, modeling $P_{c}$ as a constant is a widely adopted assumption for system-level analysis. The power consumed by each phase shifter $P_{n}(b)$ is set to 0.01 mW [66]. In Fig 3, for a typical UE located at a random distance ξ from the BS, we have compared the direct and RIS-assisted user association and outage probabilities against different values of blockage density $\lambda_{B}$ with an RIS density of $\lambda_{R}=\text{1}\text{0}\text{0}\text{0}/{km}^{\text{2}}$. The range of blockage densities has been selected considering a dense urban scenario with a moderate RIS deployment density. In case of direct UE association, $P_{d}$ monotonically decreases with increasing $\lambda_{B}$. This is due to the fact that the direct link is more affected by blockages at longer distances. In contrast, when the direct link is blocked, it can be seen that the connectivity of the UE is more dependent on the RIS reflections. As blockage density grows, the sustainable RIS-assisted association probability $P_{S-RIS}$ rises from 0.29 to 0.8 showing that even a moderate RIS deployment can achieve self-sustainable operation of enough RISs providing LoS connection to the UE. The crossover point (~1000 −1200 blockages/km²) indicates where sustainable LoS RIS links become dominant over direct links. Even though sustainable LoS RISs help maintain connection with the UE in the absence of direct link, very high blockage density still increases the chance that both direct and RIS-assisted links are blocked. This fact can be observed when the blockage density increases from 1500 blockages/km². The non-sustainable RIS-assisted link has a growing trend whereas sustainable RIS-assisted link shows a decaying trend after 2500 blockages/km². The decaying trend of sustainable RIS-assisted link probability in the highly dense blockage region is due to the fact that either farther RISs are not able to harvest enough energy for their operation or there might still be some self-sustainable RISs available for connection but they might not be able to maintain LoS with the UE due to very high density of blockages. In contrast, the growing trend of $P_{Conv}$ shows that RISs can still provide reliable connection when provided with the external power source. The performance difference of sustainable and conventional RIS links remains low even at 2000 blockages/km² which highlights the advantage of our proposed association scheme. It can also be observed that the outage probability $P_{outage}$ remains relatively low for moderate density of blockage demonstrating that RIS deployment significantly enhances coverage resilience. However, for $\lambda_{R}=\text{1}\text{0}\text{0}\text{0}/{km}^{\text{2}}$, highly dense blockage causes $P_{S-RIS}$ to saturate and $P_{outage}$ to increase.

**Fig 4:**
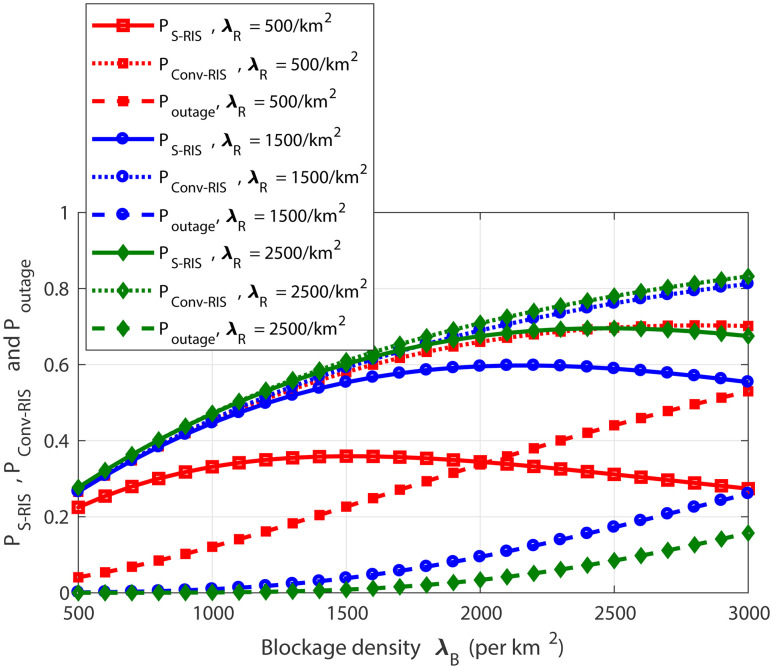
Sustainable and conventional RIS-assisted user association and outage probability vs. blockage density $\lambda_{B}$ for different values of $\lambda_{R}$.

In Fig 4, we have compared the sustainable and conventional RIS-assisted user association probabilities, $P_{S-RIS}$ and $P_{Conv-RIS}$ against different values of blockage density $\lambda_{B}$ for varying RIS deployment densities $\lambda_{R}$. The sustainable RIS-assisted association probability $P_{S-RIS}$ increases with $\lambda_{B}$ up to a certain point for all the three values of $\lambda_{R}$. When the blockage density rises from 2000 to 3000 blockages per km^2^ representing highly dense urban environment, $\;P_{S-RIS}$ saturates showing different trends for different values of $\lambda_{R}$. For $\lambda_{R}=\text{5}\text{0}\text{0}/{km}^{\text{2}}$, $P_{S-RIS}$ decays very fast. In comparison, at $\lambda_{R}=\text{1}\text{5}\text{0}\text{0}/{km}^{\text{2}}$, $P_{S-RIS}$ increases to 0.6 for moderate blockages that is 2000 blockages/km². At $\lambda_{R}=\text{2}\text{5}\text{0}\text{0}/{km}^{\text{2}}$, $P_{S-RIS}$ grows to 0.7 up to 2500 blockages/km² and then shows a decaying trend for highly dense blockages. This trend shows that with a limited RIS deployment density, there is less room for sustainable LoS operation of RISs and hence less chance for sustainability-aware user association. Meanwhile, increasing $\lambda_{R}$ provides better chances for sustainable RISs to attain a LoS link with the UE and hence provide better $P_{S-RIS}$ thereby improving network connectivity and reducing overall energy consumption of the network. The outage probability $P_{outage}$ grows gradually with $\lambda_{B}$ but, notably, it remains low even for higher $\lambda_{B}$ when RIS deployment density is large. This analysis confirms that higher RIS density seems to reduce the impact of blockages on the network and effectively mitigates outage by providing more alternative sustainable LoS reflection paths. Further, it can be observed that there is a tradeoff between RIS deployment density and sustainable operation. This tradeoff arises from the balance between availability and feasibility of RISs. Increasing the RIS density improves the probability of finding at least one RIS that satisfies both energy and LoS constraints, thereby enhancing sustainability-aware association. However, not all deployed RISs contribute effectively, as many RISs may be located in unfavorable positions with insufficient harvested energy or blocked propagation paths. As a result, the performance gain exhibits diminishing returns beyond a certain RIS density. This indicates that while higher RIS density improves sustainability, an optimal deployment density exists that balances performance gains with deployment cost and practical constraints.

**Fig 5:**
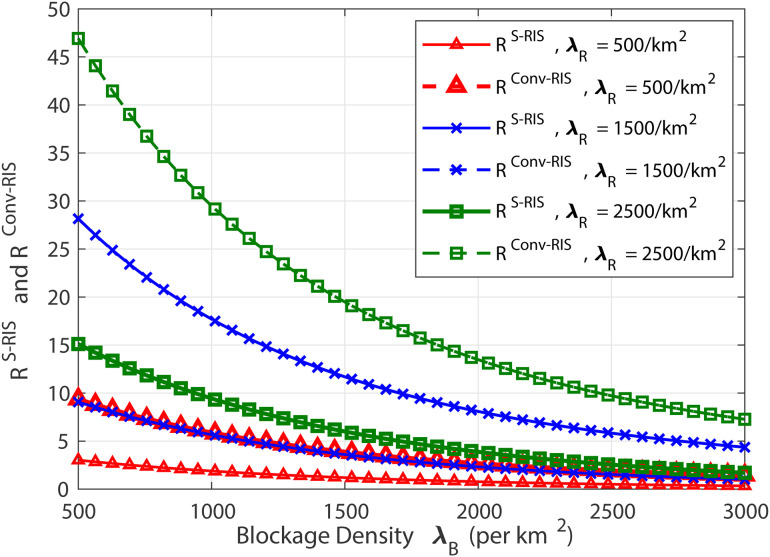
Average number of sustainable and conventional RISs vs. blockage density $\lambda_{B}$ for different values of $\lambda_{R}$.

Figure 5 illustrates the variation in the average number of sustainable LoS RISs in the cell coverage area with respect to blockage density $\lambda_{B}$ for different values of $\lambda_{R}$. The number of sustainable RISs increases with an increase in $\lambda_{R}$ even for higher values of $\lambda_{B}$. At $\lambda_{R}=\text{5}\text{0}\text{0}/{km}^{\text{2}}$, the average number of sustainable LoS RISs is 3.

Increasing $\lambda_{R}$ to $\text{1}\text{5}\text{0}\text{0}/{km}^{\text{2}}$ and $\text{2}\text{5}\text{0}\text{0}/{km}^{\text{2}}$ increases this number to 10 and 15 respectively. These results indicate that increasing $\lambda_{R}$ significantly improves sustainable operation of RISs by providing more potential reflective links, even in highly blocked environments. It means that denser RIS deployment provides a greater chance for individual RISs to harvest sufficient power from the BS, thus ensuring continuous self-sustainable operation. Conversely, very dense blockages severely limit the availability of sustainable LoS RISs. This fact is obvious from the figure, where the average number of sustainable RISs drops to 5 at $\lambda_{B}=\text{2}\text{5}\text{0}\text{0}/{km}^{\text{2}}$. Therefore, maintaining a balance between RIS deployment density and blockage density is crucial to ensure that the RISs remain self-sustainable and capable of providing reliable reflections.

**Fig 6:**
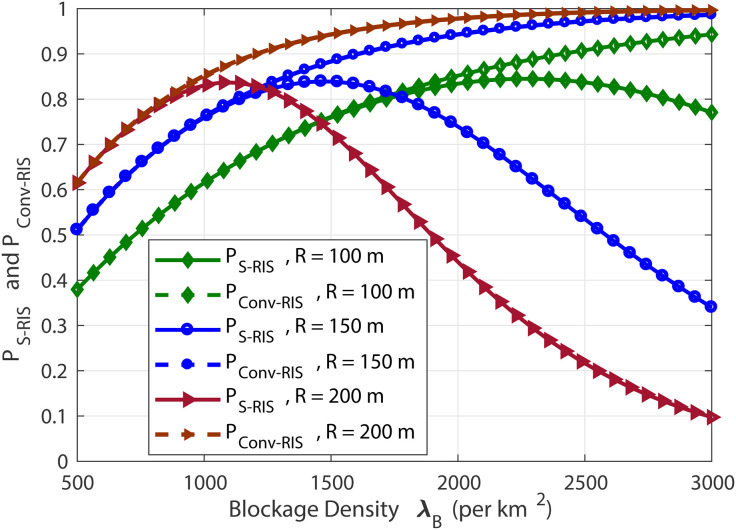
Sustainable and conventional RIS association probabilities vs. blockage density $\lambda_{B}$ for a cell-edge user with RIS density $\lambda_{R}=2500$ per km^2^ at different values ofR.

Figure 6 presents the RIS-assisted user association probability and outage probability for a cell-edge user, plotted against $\lambda_{B}$ for different values of cell radius R. A clear tradeoff is observed between link reliability and blockage density. For $\lambda_{B}<\text{1}\text{0}\text{0}\text{0}/{km}^{\text{2}}$, the RIS-assisted association probability for both sustainable and conventional case remains relatively small for a cell-edge user because the direct link between the BS and user dominates, leaving less opportunity for an RIS-assisted link. As the blockage density increases, the direct links become more frequently obstructed by blockages, and the RIS reflections begin to play a dominant role in maintaining link connectivity. At R=100 m and $\lambda_{R}=\text{2}\text{5}\text{0}\text{0}/{km}^{\text{2}}$, the values of $P_{S-RIS}$ and $P_{Conv}$ are almost same upto 2000 blockages/km^2^. At $\lambda_{B}=\text{3}\text{0}\text{0}\text{0}/{km}^{\text{2}}$, $P_{S-RIS}$ is less than $P_{Conv}$ but still achieves a value of 0.8. This result shows that a dense RIS deployment in a small cell coverage area facilitates sustainable RIS operation for a cell-edge user even at moderate to high blockage density. However, at R=150 m and 200 m, a cell-edge user is more dependent on RIS reflections but $P_{S-RIS}$ saturates as $\lambda_{B}$ increases to 900 and 1300 respectively for $\lambda_{R}=\text{2}\text{5}\text{0}\text{0}/{km}^{\text{2}}$. These results highlight that a denser RIS deployment can effectively improve sustainable RIS link reliability for a cell-edge user in highly dense urban environments.

**Fig 7:**
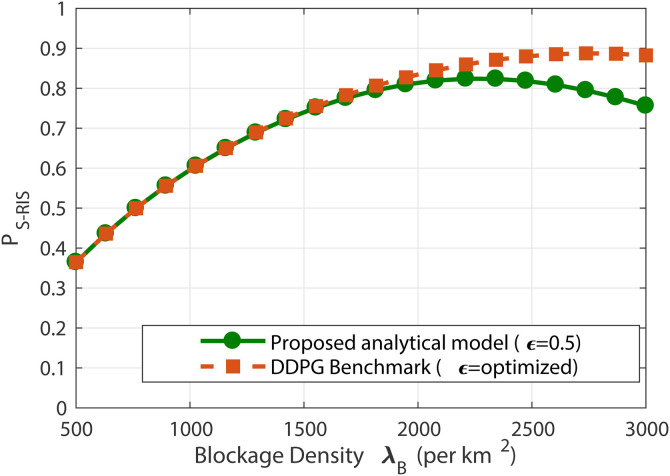
Performance comparison between the proposed model and the DDPG-based benchmark for Sustainable RIS association probability as a function of blockage density $\lambda_{B}$ assuming $\lambda_{R}=2500/{km}^{2}$.

Figure 7 illustrates the sustainable RIS association probability as a function of blockage density for the proposed sustainability-aware analytical model and the DDPG-based benchmark [34]. The DDPG framework is used to learn the optimal value of ϵ based on blockage density and RIS position. An actor–critic structure is employed in DDPG, where the actor predicts ϵ and the critic evaluates its effectiveness using a reward based on sustainability probability. After training, ϵ is adaptively selected and used to improve the association probability.

As evident from Fig 7, the proposed model depict comparable performance to that obtained through DDPG until $\lambda_{B}=\text{2}\text{0}\text{0}\text{0}/{km}^{\text{2}}$. However, at higher blockage densities, the DDPG-based approach outperforms the proposed approach. Although DDPG results in improved performance, the gain comes at the expense of significantly higher computational complexity. The analytical model completes execution in approximately 3s, whereas the DDPG framework requires around 70s. This increase in execution time is due to the iterative policy learning and soft-update processes inherent to the DDPG architecture. While the analytical model offers faster evaluation at the expense of lower value of $P_{S-RIS}$, the DDPG approach achieves superior performance with additional computational overhead, highlighting a noticeable trade-off between performance and complexity.

**Fig 8:**
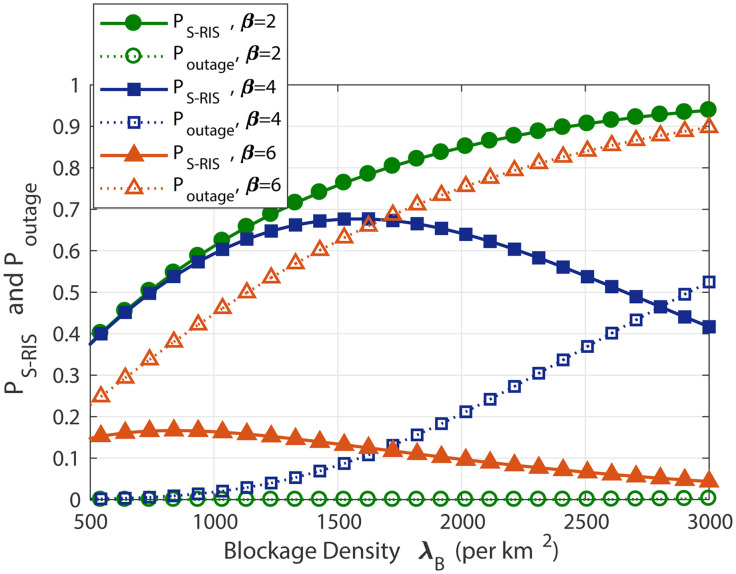
Sustainable RIS association and probabilities vs. blockage density $\lambda_{B}$ for different values of path loss exponent β with $\lambda_{R}=\text{2}500/{km}^{2}$.

Figure 8 shows the impact of path loss exponent β on sustainable RIS-assisted association and outage probability. It is observed that for β=2, $P_{S-RIS}$ remains high across all blockage densities, while outage is negligible. However, as β increases, the system experiences significant degradation due to increased signal attenuation. In particular, for β=4, a non-monotonic behavior is observed where $P_{S-RIS}$ initially improves upto $\lambda_{B}=\text{1}\text{5}\text{0}\text{0}/{km}^{\text{2}}$. At $\lambda_{B}>\text{1}\text{5}\text{0}\text{0}/{km}^{\text{2}}$, there is a significant degradation in $P_{S-RIS}$. Under severe conditions at β=6, the RIS is unable to compensate for the high path loss resulting in very low values of $P_{S-RIS}$ thereby high outage. These results highlight that the effectiveness of RIS is strongly dependent on the propagation environment.

**Fig 9:**
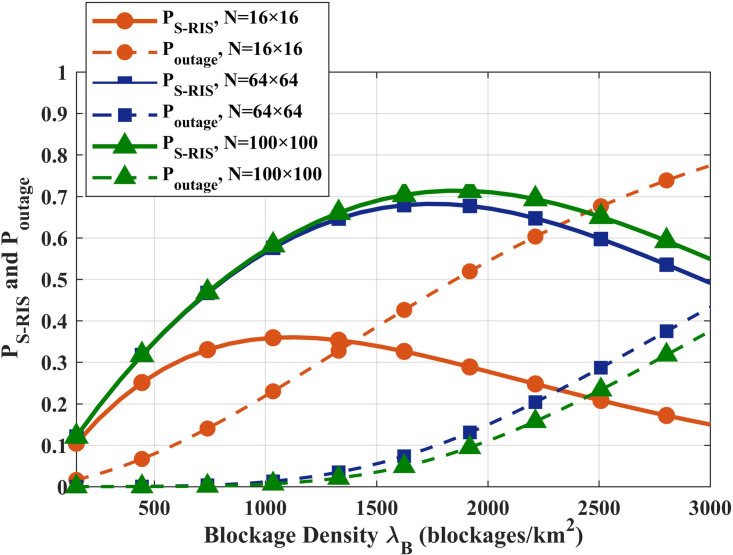
Sustainable RIS association and outage probabilities vs. blockage density $\lambda_{B}$ for different RIS element configurations with $\lambda_{R}=2500/{km}^{2}$.

Figure 9 illustrates the variation of $P_{S-RIS}$ and $P_{outage}$ with $\lambda_{B}$ for different RIS configurations. Since direct link dominates at low blockage densities, $P_{S-RIS}$ remains relatively low across all configurations. As $\lambda_{B}$ increases, RIS-assisted link becomes more effective leading to a rise in $P_{S-RIS}$ which attains peak values of approximately 0.35 for N=16×16, 0.68 for N=64×64, and 0.72 for N=100×100.

Beyond moderate blockage densities, $P_{S-RIS}(\xi )$ gradually decreases for N=64×64 and N=100×100. For N=16×16, $P_{S-RIS}$ slightly increased and started to decrease even at low to moderate values of $\lambda_{B}$ indicating insufficient energy harvesting capability for small RIS dimensions. Similarly, $P_{outage}$ increases steadily with blockage density for N=64×64 and N=100×100. However, for N=16×16, $P_{outage}$ reaches a value of 0.3 even at $\lambda_{B}=\text{1}\text{2}\text{5}\text{0}/{km}^{\text{2}}$. This analysis reveals that although large RIS size improves performance, the performance gap from 64×64 to 100×100 is relatively small. This limited performance gap arises due to diminishing returns, where increasing the number of RIS elements leads to insufficient energy harvesting causing $P_{S-RIS}$ to saturate as the system becomes constrained by path loss and blockage effects. Consequently, larger RIS panels improve link availability, but the incremental benefit reduces, especially under dense blockage conditions.

**Fig 10:**
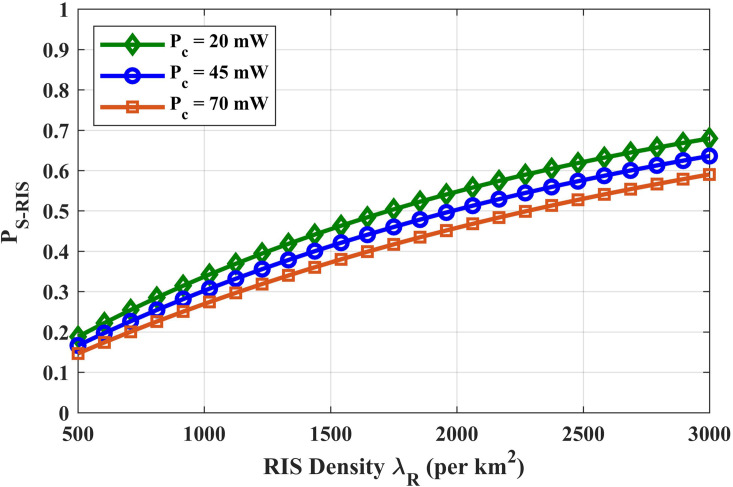
Sustainable RIS association vs. RIS density $\lambda_{R}$ for differentvalues of controller power with$\lambda_{B}=2500/{km}^{2}$.

Figure 10 shows the variation of $P_{S-RIS}$ with $\lambda_{R}$ for different values of $P_{c}$. It can be observed that $P_{S-RIS}$ increases steadily with $\lambda_{R}$, indicating the probability of establishing a reliable RIS-assisted link. However, the performance varies for different values of $P_{c}$ highlighting the impact of RIS controller power consumption.

The highest value of $P_{S-RIS}$ is achieved with $P_{c}=\text{1}\text{9}\;mW$, followed by 45 mW and 70 mW. This is because increased controller power reduces the harvested energy required for sustainable RIS operation. These results show a clear trade-off between RIS density and energy sustainability. From a practical perspective, maximizing the benefits of dense RIS deployment requires the design of low-power RIS controllers to ensure efficient and sustainable operation.

**Fig 11:**
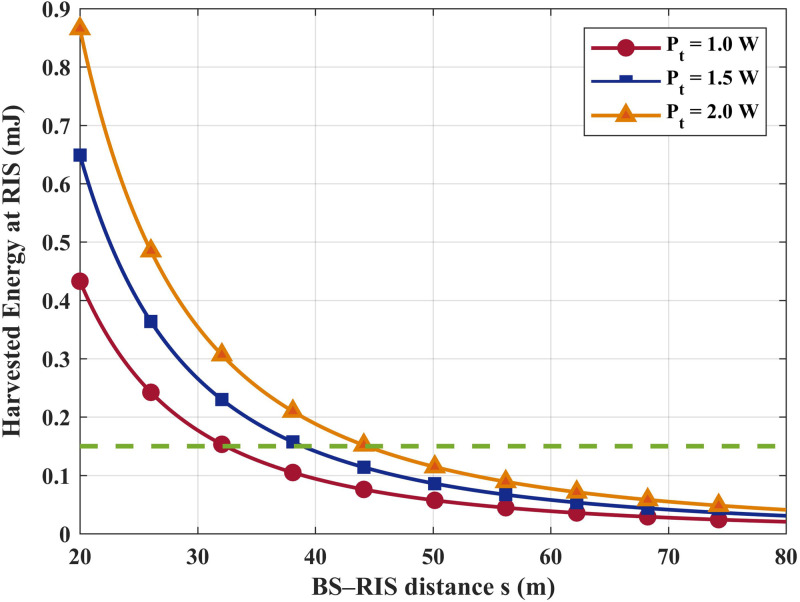
Harvested energy at sustainable RIS against BS-RIS distance for different values of BS transmit power.

Figure 11 shows the harvested energy at RIS as a function of the BS-RIS distance s for different values of BS transmit power $P_{t}$. The results are normalized per sub-array of 12 elements. This allows the sustainability threshold to be visualized at 0.14 mJ, which scales linearly to the total 140 mJ required for the full 110×110 array over a 1 s interval. The horizontal dashed line represents the minimum energy threshold required for sustainable RIS operation, clearly indicating a maximum feasible BS–RIS distance beyond which RISs become non-sustainable.

At a short BS–RIS distance of 20 m, the harvested energy is relatively high. Specifically, for $P_{t}=\text{1}\;W$, the RIS harvests approximately 0.43 mJ, which increases to about 0.65 mJ for $P_{t}=\text{1}.\text{5}\;W$ and further to nearly 0.88 mJ for $P_{t}=\text{2}\;W$. This demonstrates that increasing the transmit power significantly enhances the harvested energy when the RIS is located close to the BS. As the distance increases to 40 m, the harvested energy drops noticeably. For $P_{t}=\text{1}\;W$, the harvested energy reduces to around 0.10 mJ, while it remains higher at approximately 0.13 mJ and 0.18 mJ for $P_{t}=\text{1}.\text{5}\;W$and $P_{t}=\text{2}\;W$, respectively. At this distance, RISs connected to the BS with higher transmit power remain above the sustainability threshold. Beyond 50 m, the harvested energy for all transmit power levels approaches the minimum required threshold indicating that most RISs at these distances become non-sustainable. These observations indicate that RIS sustainability is highly sensitive to both BS transmit power and RIS placement. While increasing $P_{t}$ can extend the sustainable operating range of the RIS, the gain diminishes at larger distances. Therefore, for practical deployments of sustainable RISs, they should be placed within 30−45 m of the BS to ensure reliable energy harvesting. In this case, highly directional mmWave transmissions will enable RISs to harvest sufficient energy while maintaining strong reflected links and improving coverage in blockage-prone environments.

**Fig 12:**
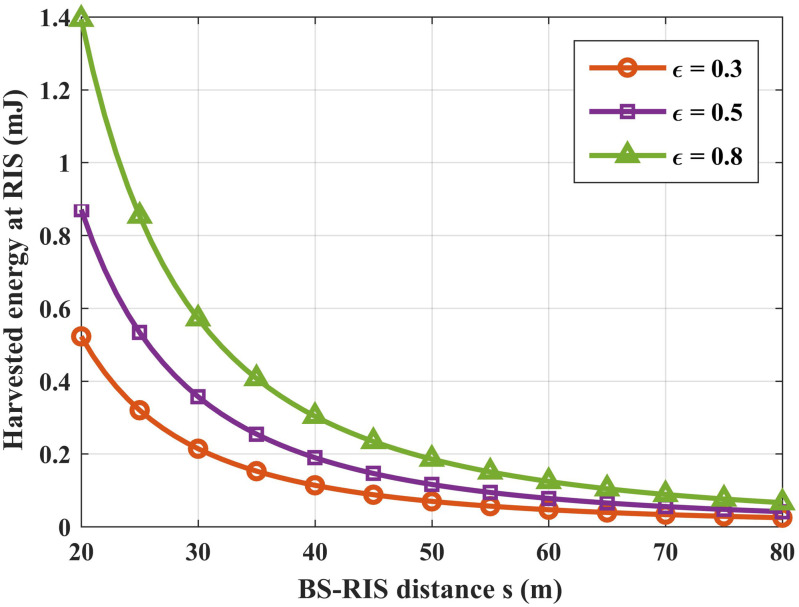
Harvested energy at sustainable RIS against BS-RIS distance for different values of power splitting ratio.

Figure 12 illustrates the variation of harvested energy at the RIS as a function of the BS–RIS distance s for different values of ϵ. It is observed that the harvested energy decreases rapidly with increasing distance, reflecting the strong impact of path loss on energy transfer. For instance, at shorter distances of around 20 m, the harvested energy is relatively high for all three values of ϵ, exceeding the threshold value of 0.15 mJ upto 35 m of BS-RIS distance.

As the distance increases, harvested energy reaches very low values beyond 65−80 m. This result highlights sustainable RIS operation is highly sensitive to the BS–RIS separation. Moreover, higher values of ϵ yield greater harvested energy, as a larger portion of the received signal is allocated to energy harvesting. However, this also implies a trade-off, since increasing ϵ may reduce the portion of power available for signal reflection. From a practical deployment perspective, the results suggest that RISs should be placed relatively closer to the BS to ensure sufficient energy harvesting for sustainable operation. Additionally, selecting an appropriate ϵ is crucial to balance energy harvesting and communication performance.

**Fig 13:**
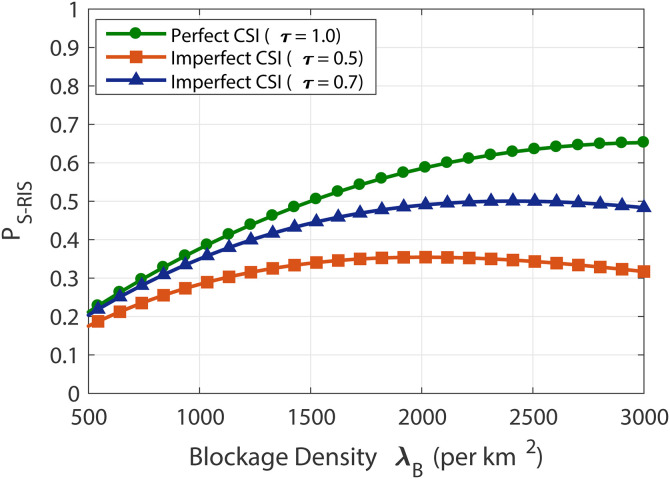
Impact of perfect and imperfect CSI on user association probability for different values of $\lambda_{B}$.

Figure 13 illustrates the impact of imperfect CSI on $P_{S-RIS}$ for a user at the centre of the cell under varying blockage densities. It is observed that the user association probability increases with blockage density for both perfect and imperfect CSI cases, indicating that RIS-assisted communication becomes increasingly beneficial in dense blockage.

At low blockage densities $\lambda_{B}=\text{5}\text{0}\text{0}/{km}^{\text{2}}$, all CSI conditions result in similar performance, with $P_{S-RIS}$ ranging from approximately 0.19 to 0.22. As blockage density increases, RIS-assisted communication becomes dominant and the performance gap for different values of CSI widens. As $\lambda_{\text{B}}$ increases above $\text{2}\text{5}\text{0}\text{0}/{km}^{\text{2}}$, $P_{S-RIS}$ with τ=1 starts to saturate whereas with imperfect CSI, $P_{S-RIS}$ shows a slightly decreasing trend indicating the importance of CSI acquisition. The sensitivity of proposed model to imperfect CSI emphasizes the need for potential extensions of the model toward adaptive and learning-assisted implementations that could be employed to improve communication reliability in practical deployment scenarios.

**Fig 14:**
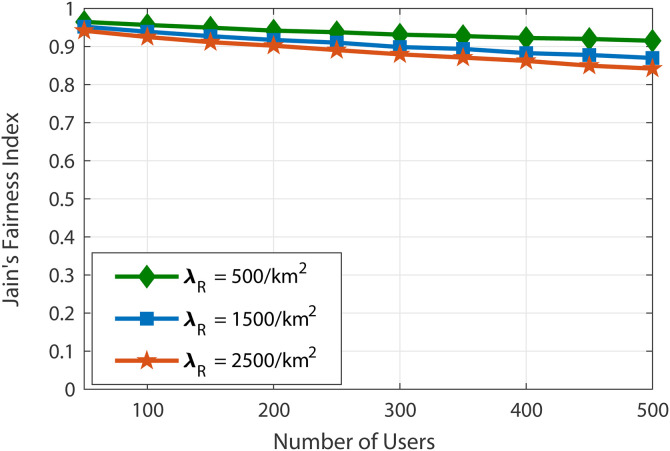
Jain’s Fairness Index against number of users for different values of λ_R._

Figure 14 shows the variation of JFI with the number of UEs for three different RIS deployment densities. The BS is assumed to be located at the origin, while UEs are uniformly distributed within a circular cell of radius 100 m. The blockage density is fixed at $\lambda_{B}=\text{3}\text{0}\text{0}\text{0}/{km}^{\text{2}}$.

The results indicate that the proposed sustainability-aware RIS-assisted framework maintains a high level of fairness across all considered deployment scenarios. For instance, at $\lambda_{R}=\text{5}\text{0}\text{0}/{km}^{\text{2}}$, the fairness index decreases slightly from approximately 0.97 at K = 50 users to 0.92 at K =500 users. This gradual decline in fairness with increasing user population is due to greater variation in achievable rates due to differences in user locations, blockage realizations, sustainable RIS availability, and channel conditions. Moreover, a slightly decaying trend in fairness is also observed as the RIS deployment density increases. Although a higher RIS density increases the number of RISs available for user association, not all deployed RISs contribute equally to network performance. Consequently, users associated with sustainable RISs in favorable locations can achieve higher rates than those relying on less sustainable RISs. This variation in RIS sustainability, combined with differences in RIS–UE distances, increases the disparity in achievable user rates and results in a slight reduction in JFI for increasing RIS density. Nevertheless, the fairness index remains above 0.84 for all considered scenarios, demonstrating that the proposed framework maintains balanced user performance under dense user deployments.

**Fig 15:**
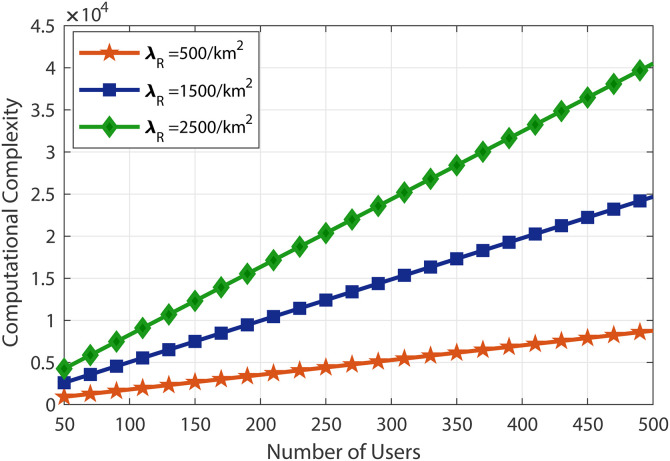
Computational complexity of the proposed framework with increasing number of users for different RIS densities.

Figure 15 shows the computational complexity of the proposed framework versus the number of UEs for different RIS deployment densities. It is observed that the computational complexity increases linearly with the user population since a larger number of users require more association decisions based on direct or S-RIS link availability. At the same time, increasing the RIS deployment density leads to a higher computational burden because more candidate RISs must be examined for each UE to ensure nearest S-RIS connection.

Nevertheless, the linear growth observed across all deployment densities demonstrates the scalability of the proposed framework with network size.

In this paper we have considered a single-cell model to ensure analytical tractability and capture the impact of sustainability-aware RIS selection on system performance. For a multi-cell network, provided that the association criteria for user remains same as considered in this work, the interference from neighboring cells will not impact the user association probability because of the distance-based association criteria in contrast to received power based criteria used in some studies. In retrospect, the interference from cells will impact signal to interference plus noise ratio (SINR) [67–70]. Hence, the sustainability-aware analytical framework may be extended to multi-cell environment with minimal impact on the analytical complexity of the model presented in this paper. Nevertheless, the primary conclusions drawn by our system model regarding the role of energy constraint and LoS conditions in RIS selection are expected to remain valid for interference limited network settings as well.

The proposed sustainability-aware framework provides useful guidance for practical RIS deployment by linking its placement to sustainability-aware user association. Results demonstrate that RISs should be positioned near base stations or dominant propagation paths with sufficient incident signal power having higher probability of LoS to users, such as elevated or less obstructed areas. Moreover, increasing RIS density improves the likelihood of finding feasible RISs that satisfy both energy and LoS constraints, although the gains may saturate beyond a certain blockage density. Hardware variability, such as differences in controller power consumption, mainly affect the energy feasibility condition but does not change the overall association behavior. Since our analysis does not account for user mobility, rapidly varying blockage conditions, or time-varying channel conditions, in highly dynamic environments, frequent updates of CSI may be required, that can impact association performance and introduce additional signaling overhead. In such scenarios, accurate CSI acquisition becomes particularly important, as our results show that imperfect CSI reduces the probability of successful UE association due to channel estimation errors. The results further indicate that the proposed association strategy maintains a balanced user-rate distribution across varying user populations and its complexity scales linearly with the number of users and sustainable RISs implying substantially lower computational overhead. proving its scalability for dense network deployments. Consequently, the proposed framework can serve as a practical guideline for RIS placement and density planning, and sustainable network design in real networks.

## IX. Conclusion

In this paper an analytical framework for deriving a sustainability-aware user association probability in an RIS-assisted mmWave network is presented. The proposed analysis captures the joint effects of blockage density, RIS deployment density, and energy sustainability on the communication link availability by incorporating the energy harvesting constraints of RISs into a stochastic geometry model. Expressions for the sustainable and conventional RIS-assisted association and outage probabilities were derived under a distance-based association criterion. The results demonstrate that increasing the RIS density significantly improves the probability of sustainable RIS operation and mitigates outage, particularly for cell-edge users in dense blockage environments. This improvement attributed to the unavailability of a direct link caused by severe blockage is compensated by a higher RIS deployment density that increases the likelihood of establishing at least one LoS reflection path through a self-sustainable RIS. Consequently, UEs that do not have access to direct link or they are located farther from the base station experience enhanced connectivity and reduced performance degradation.

However, to ensure sustainable operation, the amount of harvested energy and hence sustainable RIS user association probability at an RIS is dependent on multiple factors such as blockage density, BS transmit power, RIS power consumption, power splitting ratio and the separation between BS and RIS. An increase in RIS density enhances system performance but this improvement is constrained by the RIS power consumption. Additionally, the array size plays a crucial role in determining the energy harvesting capability of an RIS. It is observed that beyond a certain blockage density, further increases in RIS density and array size result in diminishing returns. However, RISs placed around 45 m of the BS performed well even at a higher blockage density. Therefore, the analysis reveals a trade-off among these parameters, emphasizing the importance of selection of optimal RIS density and appropriate placement in the coverage region. The proposed framework offers insight for sustainability-aware user association and RIS deployment, enabling the realization of energy-efficient, reliable, and self-sustained RIS-assisted mmWave networks in the presence of blockages. Moreover, the computational complexity of the proposed model increases linearly with increase in the number of UEs or increase in the RIS density. Furthermore, the fairness analysis of the proposed model using Jain’s fairness index indicates that all users get consistently fair share of resources which is critical for practical implementation.

Although this framework adopts distance based user association under static RIS operation, to enhance the applicability of our proposed framework to 6G networks, the framework can be extended in multiple directions. The proposed framework can be generalized to multi-cell environments by incorporating inter-cell interference and SINR-based UE association [70]. Secondly, learning-assisted approaches [71] can dynamically adapt RIS selection based on observed channel, blockage, and energy. The framework can also be extended to support cooperative multi user MIMO or distributed beamforming, along with load-aware association to balance users across RISs and improve network throughput [72,73]. While the association policy may evolve, the key insights on energy sustainability and LoS conditions in determining feasible RIS sets remain unchanged.

Additionally, dynamic RIS operation can be considered, where RIS configurations adapt to time-varying energy availability and user mobility. Advanced energy harvesting models, including non-linear and hybrid energy sources, can be integrated to better capture practical hardware behavior. Finally, data-driven or learning-based approaches can be employed to enable adaptive and scalable RIS-assisted network management in complex 6G scenarios.

## Supporting information

S1 File Data. (XLSX)
